# A severe case of cardiospondylocarpofacial syndrome with a novel *MAP3K7* variant

**DOI:** 10.1038/s41439-024-00265-0

**Published:** 2024-02-22

**Authors:** Hiromi Nyuzuki, Junichi Ozawa, Keisuke Nagasaki, Yosuke Nishio, Tomoo Ogi, Jun Tohyama, Takeshi Ikeuchi

**Affiliations:** 1 Department of Pediatrics, Niigata University Medical and Dental Hospital, Niigata, Japan; 2 Center for Medical Genetics, Niigata University Medical and Dental Hospital, Niigata, Japan; 3 Department of Pediatrics, Nagoya University Graduate School of Medicine, Nagoya, Japan; 4 Department of Genetics, Research Institute of Environmental Medicine (RIEM), Nagoya University, Nagoya, Japan; 5 Department of Child Neurology, National Hospital Organization Nishiniigata Chuo Hospital, Niigata, Japan

**Keywords:** Disease genetics, Diseases

## Abstract

Cardiospondylocarpofacial syndrome (CSCFS) is a congenital malformation characterized by growth retardation, facial features, short toes with carpal and tarsal fusion, extensive posterior neck vertebral fusion, congenital heart disease, and deafness. Here, we report a severe case of CSCFS with a novel variant, p.Thr187Ile, in *MAP3K7*. Thr187 is the main phosphorylation site for TGF-beta-activated kinase 1 encoded by *MAP3K7*, and this variant may cause significant abnormalities in downstream signaling.

Cardiospondylocarpofacial syndrome (CSCFS; OMIM#157800) is a congenital malformation syndrome characterized by growth retardation, peculiar facial features, short toes with carpal and tarsal fusion, extensive posterior neck vertebral fusion, congenital heart disease, and hearing loss with inner ear deformity. *MAP3K7*, which encodes TGF-beta-activated kinase 1 (TAK1), a phosphatase in the MAP cascade, is responsible for the autosomal dominant inheritance pattern^1–4^. Only a few cases have been reported to date, and the details of their frequency and clinical presentation are still unknown. Here, we present a case of CSCF syndrome with a novel variant of *MAP3K7*. This case exhibited a more severe phenotype than that of previously reported cases. The variant in this case was novel and located at a phosphorylation site that plays an important role in TAK1 activation, suggesting a genotype-phenotype linkage.

The patient was a girl born to healthy nonconsanguineous parents, and her 4-year-old sister was healthy. No abnormalities were noted on fetal echocardiography, and the baby was born at 40 weeks and 0 days of gestation and delivered spontaneously. Her birth weight, length, occipitofrontal circumference, and chest circumference were 2660 g (−1.35 SD), 47.0 cm (−1.32 SD), 32.5 cm (−0.69 SD), and 31.0 cm, respectively, and her Apgar score at birth was 6 for 1 min and 7 for 5 min. Characteristic facial features, including hypertelorism, a prominent forehead, full cheeks, low-set and posteriorly rotated ears, a broad nasal bridge, anteverted nares, and a long philtrum, were noted (Fig. 1a–c). She had respiratory dysfunction since birth, tachypnea, and hypoxemia. Chest radiography revealed cardiomegaly and thoracic vertebral and thoracic hypoplasia (Fig. 1d). She underwent bilateral pulmonary artery banding 4 days after echocardiography, which revealed aortic stenosis, ventricular septal defect, aortic valve stenosis, mitral regurgitation, and tricuspid regurgitation. Computed tomography (CT) and radiography showed thoracic abnormalities and extensive fusion of the cervical and thoracic vertebrae (Fig. 1e–g). In addition, she had a fragile atrioventricular valve, skin laxity, severe growth retardation, and developmental motor delay. Her chromosomal karyotype was 46,XX, and urinary mucopolysaccharide analysis and lysosomal enzyme activity assays were normal. She was suspected to have vascular Ehlers–Danlos syndrome; however, no significant variant in the responsible gene *COL3A1* was detected. Chromosomal microarray analysis did not reveal any pathological copy number variation. She underwent percutaneous angioplasty at one month of age for cardiac complications and received medications, including catecholamines, diuretics, phosphodiesterase III inhibitors, and calcium-sensitizing drugs. The patient required continuous oxygen. She exhibited marked growth retardation, with a height and weight of 57.2 cm (−6.48 SD) and 4240 g (−7.93 SD), respectively, at 12 months of age. Longitudinal growth of the thorax was also inhibited by extensive fusion of the cervical and thoracic vertebrae.

**Fig. 1:**
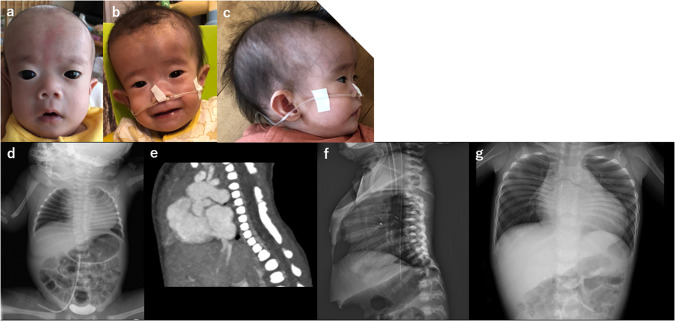
Clinical and radiological features. Facial appearance of the patient at six (**a**), 10 (**b**), and 11 months of age (**c**). Hypertelorism, a prominent forehead, full cheeks, low-set and posteriorly rotated ears, a broad nasal bridge, anteverted nares, and a long philtrum were noted. Chest radiographs taken at 1 d of age show cardiomegaly, thoracic vertebral hypoplasia, and thoracic hypoplasia (**d**). Computed tomography examination at 1 d of age and chest radiography at 19 days of age show extensive fusion at C6–T1 and T2–10 (**e**, **f**). Longitudinal growth of the thorax was also inhibited at 21 months of age (**g**).

Her psychomotor development was delayed: acquiring the ability to roll over at eight months, babbling at 9 months, sitting alone at 17 months, and pulling to stand at 19 months. Her teeth erupted at 13 months of age. Moderate hearing loss due to stenosis of the external auditory canal was observed, and she started wearing hearing aids at the age of 19 months. Rehabilitation and education were initiated at the age of 20 months to provide developmental support. She exhibited worsening hypoxemia with weight gain and underwent cardiac catheterization at 21 months of age, which revealed heart failure with a left ventricular ejection fraction of 37% and a right ventricular ejection fraction of 42%. After the examination, vomiting triggered the onset of a lethal arrhythmia, and the patient did not respond to resuscitation and died.

Whole-exome analysis was performed at eight months of age to identify the cause of the disease. Figure 2a shows the family pedigree. After obtaining written consent from the parents, genomic DNA was collected from the patient and parents, and whole-exome analysis was performed. As a result, a de novo c.560 C > T (p.Thr187Ile) heterozygous variant was detected in *MAP3K7* (NM_003188) in the patient. According to the ACMG guidelines^5^, this variant is classified as pathogenic (PS2 + PM2 + PP2-5). This variant was confirmed by Sanger sequencing and is evolutionarily conserved across multiple species (Fig. 2b, c). Together with its high consistency with the clinical picture, this case was confirmed to be CSCFS.

**Fig. 2:**
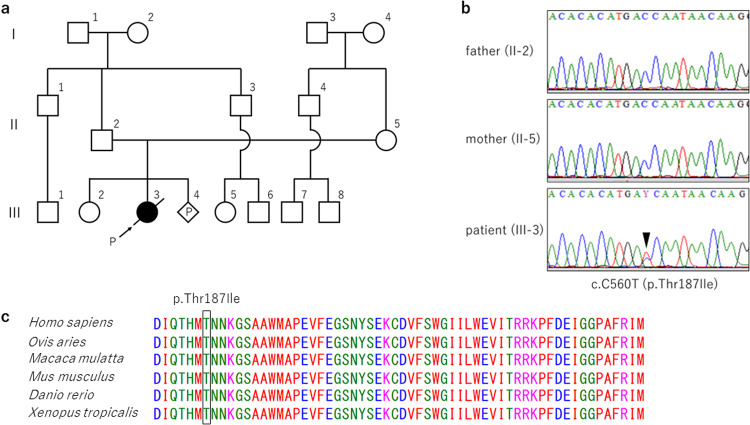
A family pedigree and genetic analysis. **a** Family pedigree of the patient. P: proband. **b** Sanger sequencing of the patient and the parents. **c** *MAP3K7* p.Thr187Ile affects an evolutionarily conserved amino acid residue in multiple species. The colors of the amino acid sequences were set according to their physicochemical properties: red for small and hydrophobic, blue for acidic, magenta for basic, and green for hydroxyl or sulfhydryl or amine.

We performed an exome analysis of a girl with multiple malformations and diagnosed her with CSCFS. To date, only nine patients with a genetic diagnosis of CSCFS have been reported^1–4^. Common symptoms include failure to thrive, characteristic facial features, skin abnormalities, hearing loss, auricular abnormalities, brachydactyly, spinal fusion, joint hypermobility, valve dysplasia, septal defects, abnormal aortic arch, poor infant feeding, and gastrointestinal dysmotility. Compared to previous cases, the clinical course of this case was more severe. The long-term prognosis of this disease is unknown; however, all cases have been reported as survivors, with the exception of an infant who died on day 9 due to diaphragmatic hernia complications, and several patients have reached adulthood. The deceased infant was a family case; the father and brother, who had the same variant in *MAP3K7*, were alive at 37 and 5 years of age, respectively^1^. In contrast, our patient had severe heart failure as a complication of CSCFS and died at one year of age. The severity of the complicated cardiac disease was a major prognostic factor, and the patient was considered to have the most severe form.

Spinal fusion has been reported in an 18-year-old male who had fusion at C2–3 and C5–7 from birth^3^ and in a 10-year-old male whose fusion at C4–5 began at 2 years of age^4^. There is also a report of a female patient with T5–6, T7–8, and T9–10 fusions at 6 years of age, followed by the appearance of C6–7 and T3–4 fusions^2^. Our case exhibited fusion at C6–T1 and T2–10 from birth, which was more extensive than that reported in previous studies. This inhibited the longitudinal growth of the thorax.

The *MAP3K7* p.Thr187Ile variant identified in this case is novel and has not been previously reported in patients with CSCFS. In vitro analysis showed that Thr187 phosphorylation plays an important role in TAK1 activity^6^. Autophosphorylation of TAK1 begins at Ser192 and proceeds in the order Thr178, Thr187, and Thr184, leading to downstream signaling. Alterations in Thr187 impair phosphorylation, resulting in abnormal downstream signaling^7–9^. It was inferred that the Thr187 variant caused significant damage to this patient. Although there are few reports of CSCFS, and the genotype-phenotype correlation is not clear, this case presented a more severe clinical picture than previously reported cases^1,2^, which may be related to the genotype.

In addition to CSCFS, *MAP3K7* is responsible for frontometaphyseal dysplasia (FMD; OMIM # 617137) and is known to have phenotypic heterogeneity. FMD is a progressive sclerosing skeletal dysplasia characterized by supraorbital hyperostosis, undermodeling of small bones, large and small joint contractures, and extraskeletal developmental abnormalities primarily of the cardiopulmonary and urinary systems^6^. The known variants of CSCFS and p.Thr187Ile found in this case are all located in the kinase domain of the *MAP3K7*. In contrast, *MAP3K7* variants in FMD2 have been reported to be located in the TAB2 binding domain in addition to the kinase domain^6^. In vitro experiments have shown that *MAP3K7* variants in FMD2 cause elevated autophosphorylation of Thr187^6^. In contrast, in CSCFS, *MAP3K7* variants have been shown to decrease TAK1 autophosphorylation and disrupt downstream TAK1-dependent signaling pathways. This difference in phosphorylation may be responsible for the phenotypic differences observed between CSCFS and FMD2^10^.

CSCF is a rare congenital disorder for which there is no effective treatment. There are many issues to be addressed in the future, such as clarification of the pathogenesis and genotype-phenotype correlation, and further accumulation of cases is desirable.

## HGV database

The relevant data from this Data Report are hosted at the Human Genome Variation Database at 10.6084/m9.figshare.hgv.3365.
